# Differences in Clinical and Imaging Features between Asymptomatic and Symptomatic COVID-19 Patients

**DOI:** 10.1155/2022/4763953

**Published:** 2022-12-14

**Authors:** Xi Ma, Zhi-Yan Lu, Yan-Juan Qu, Li-Hong Xing, Yu Zhang, Yi-Bo Lu, Li Dong, Hong-Jun Li, Li Li, Xiao-Ping Yin, Chuan-Jun Xu

**Affiliations:** ^1^CT/MRI Room, Affiliated Hospital of Hebei University, Baoding, Hebei Province 071000, China; ^2^Department of Radiology, Zhongnan Hospital of Wuhan University, Wuhan, Hubei Province 430071, China; ^3^Department of Radiology, The Fourth People's Hospital of Nanning City, Nanning, Guangxi 530023, China; ^4^Department of Radiology, Baoding People's Hospital, Baoding 071000, China; ^5^Department of Radiology, Beijing Youan Hospital, Capital Medical University, Beijing 100069, China; ^6^Department of Radiology, The Second Hospital of Nanjing, Nanjing University of Chinese Medicine, Nanjing, Jiangsu Province 210003, China

## Abstract

**Objectives:**

The clinical and imaging features of asymptomatic carriers of severe acute respiratory syndrome coronavirus 2 and symptomatic COVID-19 patients.

**Methods:**

The clinical and chest computed tomography imaging data of 47 asymptomatic carriers and 36 symptomatic COVID-19 patients were derived. All patients underwent 4–6 CT scans over a period of 2–5 days.

**Results:**

The bulk of asymptomatic carriers who developed symptoms and most of the COVID-19 patients were older than 18 years of age with a decreased lymphocyte count, abnormal hepatic and renal function, and increased D-dimer and C-reactive protein. In the early stage, the pulmonary lesion involved mostly 1–2 lobes at the peripheral area in asymptomatic carriers but more than three lobes at both the central and peripheral areas in COVID-19 patients. In the progression stage, the lesion of asymptomatic carriers extended from the peripheral to the central area, and no significant difference was found in the lesion range compared with the symptomatic control group. In early improvement stage, the lesion was rapidly absorbed, and lesions were located primarily at the peripheral area in asymptomatic carriers; contrastingly, lesions were primarily located at both the central and peripheral areas in symptomatic patients. Asymptomatic carriers reflected a significantly shorter duration from disease onset to peak progression stage compared with the symptomatic.

**Conclusions:**

Asymptomatic carriers are a potential source of transmission and may become symptomatic COVID-19 patients despite indicating less severe pulmonary damage, earlier improvement, and better prognosis. Early isolation and intervention can eliminate such carriers as potential sources of transmission and improve their prognosis.

## 1. Introduction

Since December 2019, the pandemic of the coronavirus disease of 2019 (COVID-19) has broken out, and till June 23, 2022, there were 546,755,823 coronavirus cases with 6,346,109 deaths [1–4], which was driven by the severe acute respiratory syndrome coronavirus-2 (the SARS-CoV-2) [5–7]. The transmitting mechanism is related to the viral S-protein with angiotensin-converting enzyme 2 (ACE2) receptors in the lower respiratory tract [8–12]. The SARS-Cov-2 virus has a strong person-to-person transmission capability and an epidemic doubling time of 6.4 to 31 days [13, 14]. Until now, the number of patients infected with this virus continues to grow globally, and a substantial number of infections have been underdiagnosed [6]. Confirmed COVID-19 pneumonia patients are the primary source of spread; however, asymptomatic carriers of the SARS-CoV-2 virus are also a source of transmission. The spread of the virus through asymptomatic carriers of SARS-CoV-2 has been reported in many studies [15–19].

“Asymptomatic SARS-CoV-2 carriers” refer to persons who have no clinical symptoms but have tested positive for viral nucleic acid in pharyngeal swab specimens or the positive serum-specific immunoglobulin *M* antibody of the virus [15]. There are two situations for asymptomatic infected people: first, the infected person has a positive nucleic acid test, and after 14 days of incubation period observation, there are no signs and symptoms of self-perception or clinical recognition, and it is always an asymptomatic infection state; second, the infected person tested positive for nucleic acid, and there were no signs and symptoms of self-perception or clinical recognition at the time of sampling, but then there was a certain clinical manifestation, that is, a “asymptomatic infection” stated in the incubation period [20]. It has been reported that approximately 30%–60% of COVID-19 patients show no symptoms or only have very mild symptoms; this does not, however, mean that these patients have a lower capability of spreading the virus [21]. In fact, the pandemic may have been caused by such covert infections. 10.9% of the asymptomatic carriers subsequently developed symptoms during the observation period to become confirmed cases. Close contact screening should not only be focused on patients who have developed illness but should also be extended to include asymptomatic cases during the incubation period to reduce the spread risk of SARS-CoV-2 [12]. The transmission efficiency of asymptomatic carriers was lower than that of confirmed cases, and it was shown to cause infection in 2.6% of those close contacts [22]. Moreover, patients who are infected with the SARS-CoV-2 virus will pass it on at a significant rate during the early stage of infection compared with the late stage [23]. It is thus necessary to implement early diagnosis, isolation, and treatment for asymptomatic carriers to control the infection source, cut off transmission routes, and protect the susceptible population. However, a few sets of clear data related to the current epidemic, its clinical features, and the imaging of dynamic changes among asymptomatic carriers are currently available. Accordingly, the present study was performed in conjunction with multiple centers to investigate the clinical features, imaging, and epidemic data of asymptomatic carriers to provide useful information for the management of these patients.

## 2. Materials and Methods

### 2.1. General Data

This retrospective study was approved by the Ethics Committee of the Hebei University Hospital. Between January 2020 and March 2020, all patients who were diagnosed as having been infected with SARS-CoV-2, without presenting symptoms, were enrolled in our six hospitals.

The inclusion criteria were patients who tested positive for the nucleic acid of the SARS-CoV-2 virus but without presenting COVID-19 pneumonia symptoms and with at least four times of complete CT images. The patients in the asymptomatic group were all tested positive for the nucleic acid of the SARS-CoV-2 virus but without presenting symptoms (also called “asymptomatic carriers”). While the test results of the nucleic acid of the SARS-CoV-2 virus of patients in the COVID-19 group were positive, but patients had symptomatic COVID-19 pneumonia. All of the patients in the two groups were tested positive for the nucleic acid of the SARS-CoV-2 virus, and the difference between the two groups is that the asymptomatic carriers had no COVID-19 pneumonia symptoms according to the novel coronavirus protocol (the seventh edition) [24, 25]. The COVID-19 pneumonia symptoms were as follows: fever, dry cough, fatigue, nasal congestion, runny nose, sore throat, and diarrhea; most severe patients developed respiratory distress syndrome, septic shock, metabolic acidosis, and coagulation dysfunction one week after onset; mild patients presented only with low fever, mild fatigue, and no pneumonia. The epidemic history and the typical signs of disease were extracted from medical records. All patients included were underwent multiple chest computed tomography (CT) scans, and all CT imaging data and all relevant clinical laboratory data were collected as follows: complete blood counts, D-dimer, hepatic and renal function, myocardial enzymes, and C-creative protein, according to the Novel Coronavirus protocol (the sixth edition and the seventh edition). The leukocyte count, lymphocyte count, neutrophil/lymphocyte ratio, and C-reactive protein, these tests reflect the severity of inflammation; the lymphocyte count and C-reactive protein were the clinical warning index of adults. Nearly 20% of COVID-19 patients have abnormal coagulation, and almost all severe and critically ill patients have coagulation disorders, the D-dimer was tested to prevent and treat the underlying venous thromboembolism in COVID-19 patients. Abnormal myocardial enzymes indicated myocardial damage. The abnormal myocardial enzymes responded to the degree of myocardial involvement. The liver involvement was measured by the liver function test index (alanine aminotransferase, aspartate aminotransferase, and so on). The kidney involvement was measured by the renal function test indicators (creatinine, urea, uric acid, and others).

### 2.2. Computed Tomography Scanning

Computed tomography scanning was conducted using a GE Discovery HD750 (GE Medical Systems), Philips Brilliance 64 (Philips, Haifa, Israel), or Siemens SOMATOM Perspective (Siemens, Shanghai, China) scanner. During scanning, the patient was in the supine position with the head entering first, and the scanning scope was set from the thoracic entrance to the level of the posterior costal diaphragm angle. For scanning with the GE Discovery HD750 scanner, the following parameters were adopted: a tube voltage of 120 kV, a tube current of 20–350 mA, a noise index of 18, a slice thickness of 5 mm, a 512 × 512 matrix, a pitch of 0.984 : 1, a lung-window width/level of 1,500/−500 HU, and a mediastinal window of 350/40 HU; the lung window was reconstructed on the axial plane with a slice thickness of 0.625–1.250 mm. The scanning parameters for the PHILIPS Brilliance 64 scanner included a tube voltage of 120 kV, a tube current of 50–300 mA, a 512 × 512 matrix, a pitch of 1, a lung-window width/level of 1,500/−550, a mediastinal window 350/35 HU, as well as axial plane reconstruction for the lung window. For the SOMATOM Perspective 64 scanner, the following scanning parameters were used: a tube voltage of 120 kV, an adaptive tube current (CARE Dose 4D), a detector collimation width of 64 × 0.6 mm, and high-resolution algorithm reconstruction with a reconstruction slice thickness of 1.5 mm and a slice interval of 1.5 mm.

### 2.3. Computed Tomography Imaging Analysis

Three experienced radiologists independently evaluated chest CT imaging, and agreement was facilitated through consultation when disagreements arose. Based on quartiles of patients and degree of lung involvement from day 0 to day 26 after disease onset, in one study, four stages were identified from the onset of initial symptoms: Stage-1 (0–4 days, *n* = 24); Stage-2 (5–8 days, *n* = 17); Stage-3 (9–13 days, *n* = 21); Stage-4 [23]. In another study, CT findings of COVID-19 pneumonia are recommended to be divided into three stages: early stage, advanced stage, and severe stage according to the extent of disease involvement and manifestation [26]. According to these studies, we divided the CT images of patients in this study into three stages as follows: early stage: 0–4 days; progression stage: when the CT images of the chest improved; improvement stage: when the CT images of the chest improved; the improvement stage was further divided into the early (when the lesion showed initial improvement) and late (7–14 days after the lesion reached its most serious conditions of the CT scans) stages.

Patients who did not indicate the above typical stages were included in a special group for analysis. The following features were used to describe chest CT imaging.

1. Pulmonary lesion density and interior features. Ground-glass opacity, consolidation, ground-glass opacity mixed with consolidation, intralobular and interlobular septal thickening, the appearance of fibrous stripes, pleural effusion, mediastinal lymph node enlargement, and lesion changes during the early, progression, early improvement, and late improvement stages.
2. Pulmonary lesion distribution. The involvement of pulmonary lobes and a decrease or increase in lesion numbers and scope concerning the progression and improvement stages.
3. Lesion location and scope. At the peripheral or subpleural area (involving the outer 1/3 of the lung), at the center, or at both the subpleural and central areas during the early, progression, and improvement stages.

### 2.4. Statistical Analysis

The SPSS Statistics 25.0 (IBM, Chicago, IL, USA) software program was used to conduct the statistical analysis. Measurement data were presented as the mean ± standard deviation and were tested using the analysis of variance and an independent *t*-test, whereas categorical data as frequency and tested with the chi-square test or Fisher's exact probability test. Statistical significance was set at *P* < 0.05.

## 3. Results

### 3.1. Clinical and Laboratory Tests

**Figure 1:**
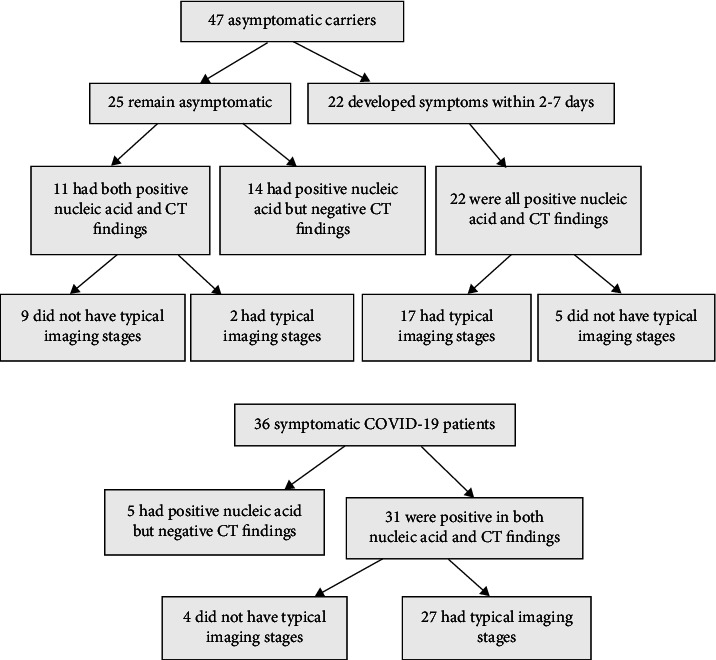
The evolution of the situation of asymptomatic carriers and symptomatic COVID-19 patients.

Forty-seven asymptomatic carriers of the SARS-CoV-2 virus were enrolled as the asymptomatic group including 30 males and 17 females with an age range of three months to 73 years (mean 37.81 ± 15.01 years) (see Figure 1 and Table 1). Concurrently, 36 symptomatic COVID-19 patients were enrolled as the control group including 25 male and 11 female participants with an age range of 6–80 years (mean 46.39 ± 16.68 years). In the control group, 13 participants had long been living in or in the nearby Wuhan area, 13 had had close contact with confirmed COVID-19 patients, and 10 had no clear epidemic history.

All 47 asymptomatic carriers had had close contact with confirmed COVID-19 patients, among which 25 cases remained asymptomatic during this study, and 22 developed clinical symptoms within 2–7 days of isolation (Figure 1). Significantly (*P* < 0.05) more patients (36%) were younger than 18 years of age among those who remained asymptomatic compared with those who developed symptoms while in isolation (4.55%) or those in the control COVID-19 group (5.56%) (see Table 1). Significantly (*P* < 0.05), more patients in the control group and among those who developed symptoms from asymptomatic carriers were older than 18 years or were elderly individuals. Concerning clinical symptoms, a sore throat had a significantly (*P* < 0.05) higher incidence (7/36, 19.44%) in the control group than among those who developed symptoms, whereas fever, cough and expectoration, headache, dizziness, fatigue, and gastrointestinal reaction were not significantly (*P* > 0.05) different between the control group and those who developed symptoms. The leukocyte and lymphocyte counts, neutrophil/lymphocyte ratio, hepatic and renal function, D-dimer, and C-reactive protein results were significantly (*P* < 0.05) greater in the control patients and those who developed symptoms compared with those who remained asymptomatic. The control group had a significantly (*P* < 0.05) higher incidence (15/36, 41.67%) of underlying diseases compared with those who remained asymptomatic (3/25, 12%) or who developed symptoms (1/22, 4.55%).

### 3.2. Computed Tomography Imaging

Among the initial 47 asymptomatic carriers, 14 showed negative CT findings related to the lungs, 19 had chest CT findings showing typical imaging stages, and 14 did not have typical imaging stages (see Figure 1). Among 36 patients with symptomatic COVID-19, five were mild infections with negative CT imaging, 27 had typical imaging stages, and four did not reflect typical CT imaging changes.

### 3.3. Pulmonary Lobe Involvement

**Figure 2:**
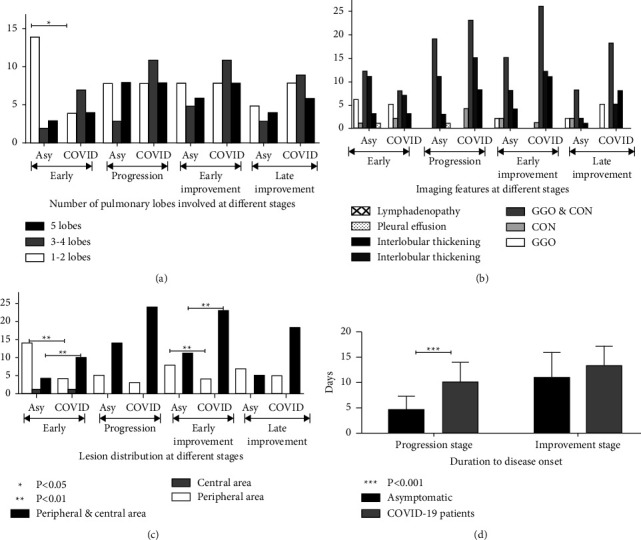
The imaging features of asymptomatic carriers (Asy) and symptomatic COVID-19 patients. (a) The pulmonary lobes that are involved at different stages of the two populations (asymptomatic carriers vs. COVID-19 patients). (b) The density of lesions in the lungs, as well as interstitial changes in the lungs of the two populations (asymptomatic carriers vs. COVID-19 patients). (c) The distribution of lesions of the two populations (asymptomatic carriers vs. COVID-19 patients). (d) The duration from progression and improvement stages to disease onset of the two populations (asymptomatic carriers vs. COVID-19 patients).  ^*∗*^GGO, ground-glass opacity; CON, consolidation.

In the early stage after disease onset, significantly (*P* < 0.05) more patients reflected one-to-two lobe involvement related to pulmonary lesions in the asymptomatic group (73.68%) compared with the symptomatic group (46.67%), which involved primarily 3–4 lobes. As the disease progressed, 3–4 (15.79%) or even 5 (42.11%) lobes were involved in the asymptomatic group (see Table 2 and Figure 2(a)). No significant (*P* > 0.05) differences were observed in the involvement of lobes at the progression and improvement stages between the asymptomatic and control groups.

### 3.4. Imaging Features

**Figure 3:**
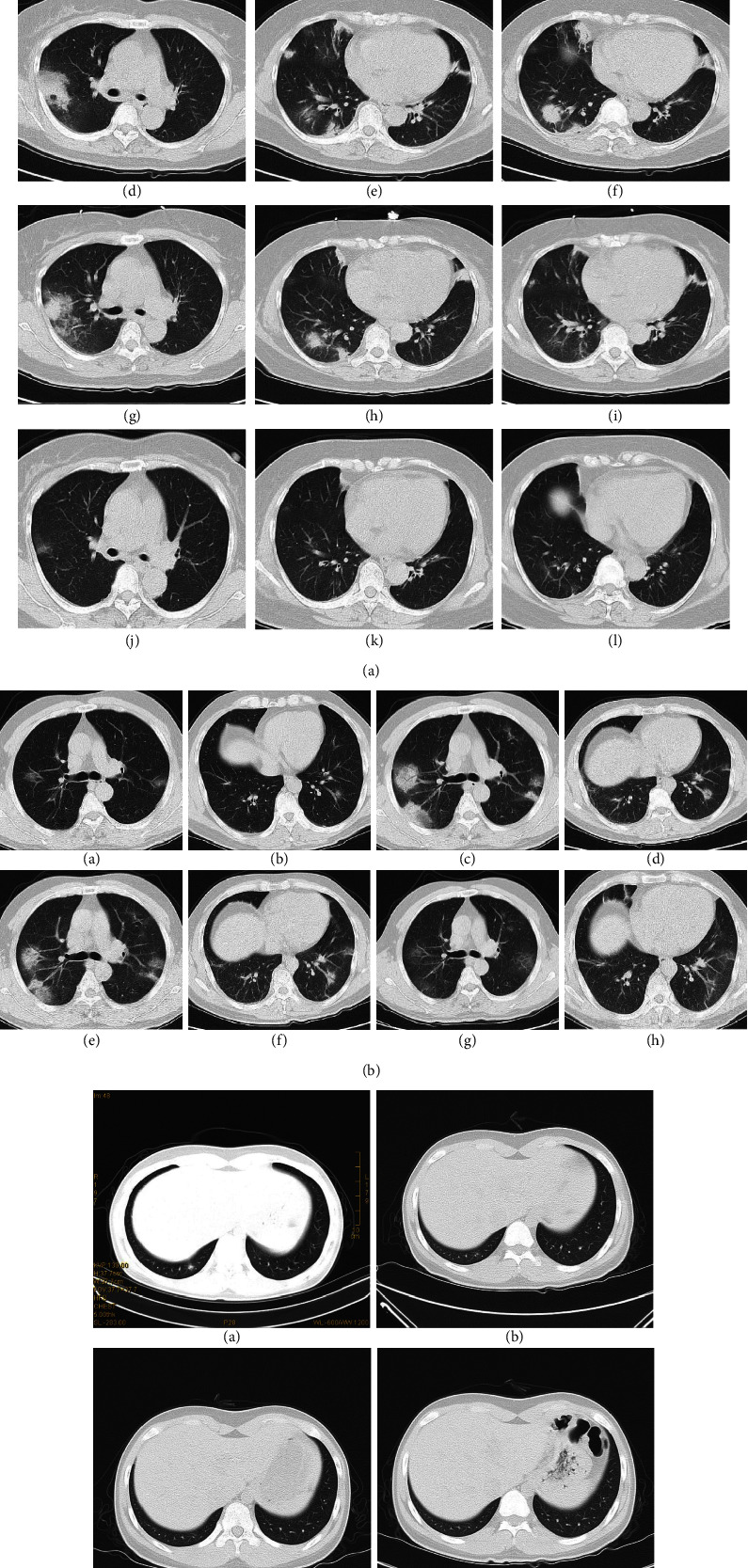
(a) Computed tomography imaging of an asymptomatic carrier who developed symptoms at a later stage. A 57-year-old woman who had hypertension for five years had close contact with a confirmed COVID-19 patient (her husband). (A) The first computed tomography imaging scan showed an area of consolidation in the right middle lobe. (B and C) Seven days later, the woman developed a slight cough; computed tomography imaging showed that the area of consolidation was enlarged in the right middle lobe. (D–F) At 11 days, new lesions developed in both the upper and lower lobes. (G–I) In the early improvement stage at 13 days, lesions in the bilateral lung area shrank and decreased in density. (J–L) The lesion in the left lung had disappeared, while the right lung lesions had been markedly absorbed. (b) Computed tomography imaging of a symptomatic COVID-19 patient. A 46-year-old man who lived in Wuhan had an intermittent cough and tested positive for viral nucleic acid. (A and B) On the day of admission, multiple patches of ground-glass opacity were observed in both upper lobes and the left lower lobe. (C and D) Four days later, computed tomography imaging showed that the lesion in both upper lobes was enlarged, and the ground-glass opacity had transformed into ground-glass opacity mixed with consolidation. An air bronchogram was observed in the right upper lobe, and new lesions appeared in both upper lobes. (E and F) In the early improvement stage, the lesions in both upper lobes shrank and reflected decreased density. (G and H) In the late improvement stage, the lesions in both upper lobes had been markedly absorbed with only patchy ground-glass opacity remaining. (c) Computed tomography imaging of a special case without symptoms (an asymptomatic carrier). A 16-year-old boy was isolated because his parents were confirmed symptomatic COVID-19 patients. (A) The first computed tomography imaging scan revealed a small high-density lesion (arrow) in the right lower lung lobe. (B) Four days later, the lesion in the right lower lobe had undergone marked shrinking (arrow). (C) At the six-day follow-up, the lesion had further shrunk and became a lesion of only 0.5 cm in diameter (arrow). (D) At the 11-day follow-up, the lesion had completely disappeared.

In the early stage of 1–4 days after disease onset, the pulmonary lesion was mostly located at the peripheral area of the lung (73.68%) in the 19 asymptomatic carriers who had typical imaging stages (see Figures 2(b), 2(c), and 3(a)), whereas the lesion was mostly located in both the peripheral and central areas (66.67%) in 15 symptomatic patients with typical imaging stages who had received CT scanning in the early stage (Table 3 and 4 and Figure 3(b)). A significant (*P* < 0.05) difference existed concerning lesion distribution in the early stage. In the progression stage, the lesion in the asymptomatic carriers extended from the peripheral to the central area and involved both the central and peripheral areas (73.68%), with no significant (*P* > 0.05) difference observed regarding lesion range compared with the symptomatic control group (88.89%) (see Table 3 and 4).

In the early improvement stage, the lesion in the asymptomatic carriers was quickly absorbed; the lesion was generally located in the peripheral area of the lung (42.11%), and there was a significant difference (*P* < 0.05) in lesion distribution compared with symptomatic patients, whose lesions primarily involved both the central and peripheral areas (85.19%). In the late improvement stage, the lesions in both the asymptomatic and symptomatic groups were absorbed, all of which started from the central to the peripheral area; no significant difference (*P* > 0.05) in the lesion distribution between the two groups was observed. No significant difference (*P* > 0.05) was observed in the imaging features of ground-glass opacity, consolidation, and ground-glass opacity mixed with consolidation or intralobular and interlobular septal thickening between the two groups. One asymptomatic carrier had pleural effusion in the early stage, and no mediastinal lymphadenopathy nor pleural effusion occurred in the progression and improvement stages of both groups (Tables 2–4).

### 3.5. Comparison of the Characteristics of the Pathological Outcome and the Time of the Progressive Outcome

In the improvement stage, the imaging features of both the asymptomatic carriers and symptomatic patients were reduced lesion extent and decreased lesion density. The symptomatic patients showed a more significant (*P* < 0.05) reduction in the lesion extent (88.89%) compared with asymptomatic carriers (52.63%). No significant (*P* > 0.05) difference was found in the decrease of lesion density between the two groups. The lesion number did not reflect significant changes in the improvement stage. The duration time from disease onset to the progression peak was significantly shorter (*P* < 0.05) in the asymptomatic carriers (4.63 ± 2.74 days) compared with the symptomatic patients (10.07 ± 3.90 days), and at the same time, the seriousness of the disease progressed faster in the asymptomatic carriers than in the symptomatic patients, but the progression of the seriousness of the disease showed no statistical significance (*P* > 0.05) (see Table 5–7 and Figure 2(d)).

### 3.6. Special Cases

Among 33 asymptomatic carriers with positive pulmonary CT findings, 14 (42.42%) did not have typical imaging stages during the disease course including 10 cases who remained asymptomatic and four who developed symptoms later. The imaging manifestations of pulmonary lesions reached peaked at the first time of CT scanning; however, the lesion was shown to have been gradually absorbed during follow-up CT scans (Figure 3(c)). Among 31 symptomatic COVID-19 patients with positive CT findings, four (12.90%) did not present the typical imaging stages. In two patients, some of the pulmonary lesions were aggravated while others had been absorbed and improved. In the other two patients, new pulmonary lesions had developed, or the original lesion was aggravated at the improvement stage.

## 4. Discussion

Coronavirus 2019-related pneumonia caused by the SARS-CoV-2 virus is currently rampant worldwide. With the spread and occurrence of intergeneration changes in the virus, the clinical manifestations of patients with COVID-19 have gradually changed, and the initial symptoms of infection have become more covert. Although some patients do not present obvious discomfort or symptoms, they are as contagious as symptomatic COVID-19 patients. It has been shown that when the clinical symptoms are still mild following infection with COVID-19, the virus will replicate very actively in the pharynx and reach a peak concentration of five days after disease onset. COVID-19 requires a much shorter time than the SARS virus to reach a peak concentration at the early stage which is 1,000 times that of the SARS virus [10]. Consequently, it is crucial that infections should be prevented, and asymptomatic patients should be treated to stop the global spread of the virus.

### 4.1. Clinical Features

In this study, asymptomatic virus carriers received medical care and subsequently returned positive nucleic acid tests because they had all had close contact with confirmed COVID-19 cases. Among these patients, 46.81% subsequently developed symptoms, while 53.19% remained asymptomatic during the course of this study. The proportion of people younger than 18 years of age among those who remained asymptomatic was significantly higher than those who developed symptoms or the number of symptomatic patients in the control group. Younger patients will typically have a stronger immune reaction and resistance against invading viruses compared with older patients, in whom immunosenescence may not allow the patient to produce a strong immune reaction and resistance against a novel virus [8]. Moreover, comorbidities may also play a role in decreasing the body's immune reaction and resistance against a virus [27]; the COVID-19 group had more comorbidities than asymptomatic patients and may have been more prone to infection and presenting with symptoms. In our study, patients who developed symptoms and patients in the control group had significantly decreased lymphocyte counts but a significantly increased ratio of neutrophils-to-lymphocytes compared with those who remained asymptomatic. Lymphocyte damage may be an important factor leading to the deterioration of COVID-19 patients' condition [27]. The ratio of neutrophils-to-lymphocytes is an independent risk factor of severe illness, particularly in the COVID-19 early stage. [28–30] Therefore, the timely detection of lymphocytes may help to better understand a patient's condition. It should be noted that the above discussion is only our conjecture based on the existing results. The conclusion of immunologic backgrounds leading to asymptomatic or symptomatic disease still lacks solid evidence to support it, and further research is needed.

In this study, abnormal hepatic and renal functioning was also observed in those who developed symptoms, and in the control symptomatic patients, D-dimer and C-reactive protein were significantly higher than in those who remained asymptomatic. Once the asymptomatic carriers developed symptoms, it indicated that the body's resistance had decreased and that the virus was able to bind to human receptors ACE2. [31] The ACE2 receptors are expressed not only in the lower respiratory tract but also in the myocardium, as well as in the liver and kidneys; the binding of the virus to the receptor consequently causes the relevant symptoms and abnormal functioning of the organs. Concurrently, the inflammatory response and C-reactive protein increased. The degree of myocardial, liver, and kidney involvement was lower in those who remained asymptomatic compared with those who developed symptoms or in the symptomatic patients, which may have been one of the reasons that those who remained asymptomatic had quicker recovery and shorter hospitalization.

### 4.2. Imaging Features

In the early stage, the pulmonary lesions involved mostly one to two lobes at the peripheral area of the lung among asymptomatic carriers but more than three lobes at both the central and peripheral areas among symptomatic patients. This indicated that fewer lobes and areas were involved in the asymptomatic carriers with most lesions at the peripheral or subpleural area, which is in line with the existing research. [32] In the progressive stage, the scope of ground-glass opacities increased and extended towards the central area; however, no significant differences were found in the areas of involvement between the asymptomatic and symptomatic groups. In the early improvement stage, the lesion was quickly absorbed, starting from the central towards the peripheral area; the lesion was primarily located in the peripheral area (42.11%) in asymptomatic carriers. However, among the COVID-19 patients in this stage, the lesion was absorbed relatively slowly and was located primarily in both the central and peripheral areas (85.19%), and there was a significant difference in the lesion distribution between the two groups. In the late improvement stage, no significant difference was observed in lesion distribution.

The pulmonary lesion was viral pneumonia involving lung parenchyma and interstitial inflammation at all stages in both groups, with no significant differences between them. In the early stage, the lesion presented primarily as having ground-glass opacity or ground-glass opacity mixed with consolidation, with accompanying intralobular or interlobular septal thickening forming a “crazy paving” sign. This pathology was caused by the virus invading the pulmonary interstitial, leading to edema and thickening of the interlobular, subpleural, central, and peribronchovascular stroma. Consolidation represents further infiltration of the parenchyma because the infection causes obvious shedding of alveolar epithelial cells, the formation of pulmonary hyaline membrane, exudation of alveolar fibrous cords, and inflammation of the alveolar septum, resulting in increased lung density. [33].

In this study, one asymptomatic carrier had pleural effusion in the early stage, which had likely been caused by inflammation involving the pleura and resulting in pleural reactive inflammation. Pleural effusion was absorbed during follow-up. No mediastinal lymphadenopathy occurred in either of the two groups.

The improvement stage was characterized by a decreased scope and density of the lesion, and COVID-19 patients showed a more significant decrease in the scope of lesions compared with asymptomatic carriers (88.89% vs. 52.63%, respectively). The asymptomatic carriers had a significantly shorter duration from disease onset to the progression peak stage compared with COVID-19 patients (4.63 ± 2.74 vs. 10.07 ± 3.90 days, respectively), and the asymptomatic carriers also had earlier improvement compared with COVID-19 patients but with no significant differences, suggesting that asymptomatic carriers typically had a short disease course with both fast progression and quick improvement.

### 4.3. Special Cases

Among 33 asymptomatic carriers with positive pulmonary CT imaging, 14 (42.42%) did not have typical imaging presentations. On the first CT scanning instance after admission, the peak progression presentations of the lesion were presented on CT imaging; in a later follow-up, the lesion had gradually improved or had been absorbed completely. This was in agreement with virological findings indicating that patients with mild symptoms at the early stage had peak nucleic acid concentrations. [10] Following the presentation of pneumonia symptoms, the virus concentration decreased, and the patient gradually recovered with complete restoration of pulmonary CT imaging. Four COVID-19 patients also had atypical CT imaging presentations in which some lesions were absorbed, while others were aggravated, indicating continuous infiltration of the virus in the lung, causing repeated pulmonary damage. In general, asymptomatic carriers had a shorter disease course and better prognosis than symptomatic patients.

Some limitations may exist in this research as follows: firstly, due to the limited cases in China recently, it is a small cohort of both asymptomatic and symptomatic participants, resulting in some bias in the research results, especially lacks the restriction of the patients' age and the matched patients between these two groups, which thus cannot fully reflect all the clinical and imaging characteristics of asymptomatic and symptomatic COVID-19 patients and may cause some selection biases; secondly, the retrospective nature of this study may also have caused some bias in its outcome; thirdly, the long-term infectiveness of asymptomatic carriers was not established beyond the end date of this study and should be further investigated; fourthly, we did not pay attention to the dose of radiation of patients, and this is a multicenter study, and every hospital used different machines and different scanning conditions, so the radiation dose was different; fifthly, the control group used in this study is composed of participants who had symptomatic COVID-19 pneumonia and presented with clinical symptoms of COVID-19 following infection, and it would be worthwhile to add another control group composed of uninfected patients to have a base reference for pulmonary lesion distribution and localization which may call for further study; sixthly, all patients are infected by the wildtype variant, which nowadays does not play a role in the pandemic anymore, and clinical features and especially severity vary between variants, thus the data seem outdated.

In conclusion, asymptomatic carriers may be a potential source of transmission and may develop into symptomatic COVID-19 patients despite having less severe pulmonary damage, earlier improvement, and an overall better prognosis. It would be better to identify asymptomatic carriers who do not have sufficient cause for testing before the presentation of clinical symptoms to eliminate being potential sources of transmission and to improve their prognosis. Both the clinical and imaging data were significant in the identification and management of asymptomatic carriers.

## Figures and Tables

**Table 1:** Clinical data of the patients [*n* (%)].

Items	Asymptomatic carriers		COVID-19 patients (*n* = 36)	*χ* ^2^	*P*
	Remain asymptomatic (*n* = 25)	Developed symptomatic (*n* = 22)			
Gender				0.64	0.73
Male	15 (60.0)	15 (68.18)	25 (69.44)		
Female	10 (40.0)	7 (31.82)	11 (30.56)		

Age				12.42	0.02
<18 years	9 (36.0)	1 (4.55)	2 (5.56)		
18–50 years	11 (44.0)	14 (63.64)	22 (61.11)		
≥50 years	5 (20.0)	7 (31.82)	12 (33.33)		

Epidemic history				52.32 ^*∗*^	<0.001
In Wuhan and surrounding areas	0	0	13 (37.14)		
Close contact	25 (100.0)	22 (100.0)	12 (33.33)		
Unclear epidemic	0	0	10 (27.78)		

Maximum body temperature				1.47 ^*∗*^	0.72
≤37.2°C		3 (13.64)	3 (8.33)		
37.3–38°C		7 (31.82)	15 (41.67)		
38.1–38.9°C		3 (13.64)	10 (27.78)		
≥39°C		1 (4.55)	3 (8.33)		

Cough		7 (31.82)	16 (44.44)	0.91	0.34

Expectoration		3 (13.64)	8 (22.22)	0.66	0.51

Sore throat		0	7 (19.44)	4.87 ^*∗*^	0.03

Headache and dizziness		1 (4.55)	0	1.67 ^*∗*^	0.38

Fatigue		7 (31.82)	4 (11.11)	3.81	0.08

Muscle ache		2 (9.1)	1 (2.78)	1.11 ^*∗*^	0.55

Chest tightness and dyspnea		1 (4.55)	2 (5.56)	0.03 ^*∗*^	1.00

Gastrointestinal reaction		0	1 (2.78)	0.62 ^*∗*^	1.00

Normal or decreased leukocyte count	13 (52.0)	22 (100.0)	31 (86.11)	18.25	<0.001

Decreased lymphocyte count	3 (12.0)	6 (27.27)	15 (41.67)	6.36	0.04

Neutrophil/lymphocyte ratio	2.03 ± 2.00	3.32 ± 1.43	4.32 ± 4.38	3.82 ^#^	0.02

Increased C-reactive protein	4 (16.0)	16 (72.73)	29 (80.56)	27.75	<0.001

Abnormal hepatic function	2 (8.0)	5 (22.73)	19 (52.78)	14.78	0.001

Abnormal renal function	2 (8.0)	5 (22.73)	13 (36.11)	6.41	0.04

Increased D-dimer	3 (12.0)	11 (50.0)	4 (11.11)	14.14	0.001

Abnormal myocardial enzymes	6 (24.0)	7 (31.82)	16 (44.44)	2.84	0.24

With comorbidities	3 (12.0)	1 (4.55)	15 (41.67)	13.06	0.001

Hospitalization days	12.08 ± 5.59	14.29 ± 5.83	15.17 ± 7.05	1.79 ^#^	0.17

No. of severe patients	0	1 (4.55)	4 (11.11)	3.33	0.17

*Note.* Asymptomatic, those who remained asymptomatic; symptomatic, those who developed symptomatic later; VOCID-19, symptomatic COVID-19 patients.  ^*∗*^The Fisher exact test was used; #ANOVA was used.

**Table 2:** Pulmonary lobes involved by lesions [*n* (%)].

Classification		No of lobes involved			*χ* ^2^	*P*
		1–2	3–4	5		
Early stage					8.12	0.01
	Asymptomatic (*n* = 19)	14 (73.68)	2 (10.53)	3 (15.79)		
	COVID-19 (*n* = 15)	4 (26.67)	7 (46.67)	4 (26.67)		

Progression and peak stages					3.28	0.19
	Asymptomatic (*n* = 19)	8 (42.11)	3 (15.79)	8 (42.11)		
	COVID-19 (*n* = 27)	8 (29.63)	11 (40.74)	8 (29.63)		

Early improvement stage					1.18	0.55
	Asymptomatic (*n* = 19)	8 (42.11)	5 (26.32)	6 (31.58)		
	COVID-19 (*n* = 27)	8 (29.63)	11 (40.74)	8 (29.63)		

Late improvement stage					0.71	0.74
	Asymptomatic (*n* = 12)	5 (41.67)	3 (25.0)	4 (33.33)		
	COVID-19 (*n* = 23)	8 (34.78)	9 (39.13)	6 (26.09)		

*Note.* Asymptomatic, those who remained asymptomatic; COVID-19, symptomatic COVID-19 patients.

**Table 3:** Imaging of asymptomatic carriers and COVID-19 patients in the early stage [*n* (%)].

CT features	Early stage		*χ* ^2^	*P*
	Asymptomatic (*n* = 19)	COVID-19 (*n* = 15)		
Prevalent lesion distribution within lobes				
Peripheral area	14 (73.68)	4 (26.67)	7.44	0.006
Central area	1 (5.3)	1 (6.67)	0.03 ^*∗*^	1.000
Both peripheral and central areas	4 (21.05)	10 (66.67)	7.20	0.007
Lesion density and interior features				
Ground glass opacity	6 (31.58)	5 (33.33)	0.01	1.000
Consolidation	1 (5.3)	2 (13.33)	0.68	0.57
Both ground-glass opacity and consolidation	12 (63.16)	8 (53.33)	0.33	0.56
Intralobular septal thickening	11 (57.89)	7 (46.66)	0.42	0.52
Interlobular septal thickening	3 (15.79)	3 (20.0)	0.10	1.00
Other features			0.71 ^*∗*^	1.00
Pleural effusion	1 (5.3)	0		
Enlarged mediastinal lymph nodes	0	0		

*Note.* Asymptomatic, those who remained asymptomatic; COVID-19, symptomatic COVID-19 patients;  ^*∗*^the fisher exact test was used.

**Table 4:** Imaging of asymptomatic carriers and COVID-19 patients in the progression stages [*n* (%)].

CT features	Progression and peak stage		*χ* ^2^	*P*
	Asymptomatic (*n* = 19)	COVID-19 (*n* = 27)		
Prevalent lesion distribution within lobes				
Peripheral area	5(26.32)	3(11.11)	1.80	0.25
Central area	0	0	—	—
Both peripheral and central areas	14(73.68)	24(88.89)	1.80	0.25
Lesion density and interior features				
Ground glass opacity	0	0	—	—
Consolidation	0	4(14.81)	3.08	0.13
Both ground glass opacity and consolidation	19(100.0)	23(85.19)	3.08	0.13
Intralobular septal thickening	11(57.89)	15(55.56)	0.02	0.88
Interlobular septal thickening	3(15.79)	8(61.54)	1.18	0.32
Other features				
Pleural effusion	1(5.3)	0	1.45 ^*∗*^	0.41
Enlarged mediastinal lymph nodes	0	0	—	—

*Note*. Asymptomatic, those who remained asymptomatic; COVID-19, symptomatic COVID-19 patients;  ^*∗*^the fisher exact test was used.

**Table 5:** Imaging features of asymptomatic carriers and COVID-19 patients at the early improvement stage.

CT features	Early improvement stage		*χ* ^2^	*P*
	Asymptomatic (*n* = 19)	COVID-19 (*n* = 27)		
*Prevalent lesion distribution within lobes*				
Peripheral area	8 (42.11)	4 (14.81)	4.31	0.04
Central area	0	0	—	—
Both the peripheral and central area	11 (57.89)	23 (85.19)	4.31	0.04

*Density and interior features*				
Ground glass opacity	2 (10.53)	0	2.97 ^*∗*^	0.17
Consolidation	2 (10.53)	1 (3.70)	0.85	0.56
Both ground-glass opacity and consolidation	15 (78.95)	26 (96.30)	3.47	0.14
Intralobular septal thickening	8 (42.11)	12 (44.44)	0.02	0.88
Interlobular septal thickening	4 (21.05)	11 (40.74)	1.97	0.16

*Other features*				
Pleural effusion	0	0	—	—
Enlarged mediastinal lymph nodes	0	0	—	—

**Table 6:** Imaging features of asymptomatic carriers and COVID-19 patients at the late improvement stage [*n* (%)].

CT features	Late improvement stage		*χ* ^2^	*P*
	Asymptomatic (*n* = 12)	COVID-19 (*n* = 23)		
Prevalent lesion distribution within lobes				
Peripheral area	7(58.33)	5(21.73)	4.69	0.06
Central area	0	0	—	—
Both peripheral and central area	5(41.67)	18(78.26)	4.69	0.06
Density and interior features				
Ground glass opacity	2(16.67)	5(21.74)	0.13	1.00
Consolidation	2(16.67)	0	4.07 ^*∗*^	0.11
Both ground glass opacity and consolidation	8(66.67)	18(78.26)	0.56	0.69
Intralobular septal thickening	2(16.67)	5(21.74)	0.13	1.00
Interlobular septal thickening	1(8.33)	8(34.78)	2.89	0.12
Other features				
Pleural effusion	0	0	—	—
Enlarged mediastinal lymph nodes	0	0	—	—

*Note*. Asymptomatic, those who remained asymptomatic; COVID-19, symptomatic COVID-19 patients; CT, computed tomography;  ^*∗*^the fisher exact test was used.

**Table 7:** Improvement of asymptomatic carriers and COVID-19 patients.

CT imaging	Improvement		*χ* ^2^	*P*
	Asymptomatic (*n* = 19)	COVID-19 (*n* = 27)		
Reduced lesion extent	10 (52.63)	24 (88.89)	7.60	0.008
Reduced lesion number	0	0	—	—
Reduced lesion extent and number	7 (36.84)	3 (11.11)	4.34	0.07
Decreased lesion density	14 (73.68)	21 (77.78)	0.10	1.00

*Note.* Asymptomatic, those who remained asymptomatic; COVID-19, symptomatic COVID-19 patients; CT, computed tomography.

## Data Availability

We declared that materials described in the manuscript, including all relevant raw data, will be freely available to any scientist wishing to use them for noncommercial purposes, without breaching participant confidentiality.

## Abbreviations

COVID-19: Corona virus disease 2019
CT: Computed tomography
The SARS-CoV-2: The severe acute respiratory syndrome coronavirus-2
ACE2: Angiotensin-converting enzyme 2.

## Additional Points

(1) Differences in imaging features between asymptomatic and symptomatic COVID-19 patients. (2) Asymptomatic patients are as contagious as symptomatic COVID-19 patients. (3) It is crucial that infections should be prevented and asymptomatic patients.
